# Use of a meta-analysis to assess the preventive effect of dexmedetomidine on cardiac surgery-associated acute kidney injury

**DOI:** 10.1186/s13054-017-1853-4

**Published:** 2018-03-27

**Authors:** Gui-Zhen Yang, Fu-Shan Xue, Ya-Yang Liu

**Affiliations:** Department of Anesthesiology, Plastic Surgery Hospital, Chinese Academy of Medical Sciences and Peking Union Medical College, 33 Ba-Da-Chu Road, Shi-Jing-Shan District, Beijing, 100144 People’s Republic of China

Shi and Tie [1] concluded in their meta-analysis that dexmedetomidine might be a promising prevention strategy for cardiac surgery-associated acute kidney injury (CSA-AKI). In a meta-analysis, the results from many studies are synthesized mathematically by complex statistical methods to assess the diversity among results and to estimate a common pooled effect with increased precision. Thus, the results of a meta-analysis are only as good as the quality of the collected data. We noted that some defects of the studies included in this meta-analysis would have made interpretation of their conclusions questionable.

First, there is a high heterogeneity among seven included studies, such as studied subjects (pediatric and adult patients), definitions of primary outcomes (creatinine rise, biomarkers, and renal complications), intervention times (unclear, intraoperative, intraoperative and postoperative, and postoperative), doses of dexmedetomidine, and so on.

Second, four of seven included studies were observational studies with significant methodological limitations and a number of confounders, such as a retrospective design or single-center recruitment. There was no attempt in some studies to control most of the risk factors for CSA-AKI, including intraoperative transfusions, hemodynamic instability, use of vasopressors, hemodilution anemia, and so on [2, 3].

Third, most of included studies did not assess the effect of dexmedetomidine on the severity and duration of CSA-AKI, although these have highly been associated with postoperative outcomes [4].

Finally, this analysis did not include the recent randomized controlled trial (RCT) by Zhai et al. [5], in which dexmedetomidine decreased the incidence and severity of CSA-AKI in patients undergoing cardiac surgery. The findings of Zhai et al. support the conclusion of this meta-analysis that dexmedetomidine may be beneficial for prevention of CSA-AKI.

## Abbreviations

CSA-AKI: Cardiac surgery associated-acute kidney injury
RCT: Randomized controlled trial
RR: Relative risks
CI: Confidence interval
