# Supplementary material for: Use of a meta-analysis to assess the preventive effect of dexmedetomidine on cardiac surgery-associated acute kidney injury
Source: Crit Care. 2018 Mar 27;22:85. doi: 10.1186/s13054-017-1853-4 (PMC5870748; doi:10.1186/s13054-017-1853-4)
Supplement: Related texts

# Reply: Authors’ response

**Authors:** Rui Shi, Hong-Tao Tie

We appreciate the commentary by Yang et al. on our recent publication [1], and we would like to provide a deep discussion according to their concerns.

Inevitably, the studies combined together in a meta-analysis differ. Statistical heterogeneity consists of clinical heterogeneity (variability in participants, interventions, and outcomes) and methodological heterogeneity (variability in study design) [6].

In our study, we separately pooled cohorts and RCTs to mitigate the influence of methodological heterogeneity. Additionally, although there was no significant heterogeneity in meta-analyses of RCTs or cohorts (RCTs, *P*_H_ = 0.52, *I*^2^ = 0%; cohort, *P*_H_ = 0.58, *I*^2^ = 0%), a random-effects model was used to reduce the impact of both clinical and methodological heterogeneity.

A retrospective cohort by Turan et al. [7] failed to show a benefit of dexmedetomidine on CSA-AKI, and the possible explanation from clinical heterogeneity might be as follows: the different criteria of CSA-AKI, defined as dialysis and anuria, might lead to turbulence of the results. Additionally, a renoprotective effect induced by selective α2-adrenoreceptor inhibition was only present for mild to moderate doses of dexmedetomidine [8], but in the current study the doses and duration of the dexmedetomidine regimen were unclear. Furthermore, CSA-AKI was the secondary outcome in the current cohort. Clinical heterogeneity could also explain the inconsistent effect of dexmedetomidine in RCTs. One RCT [8] reported a null effect of dexmedetomidine on CSA-AKI according to RIFLE classification; preventive effects of dexmedetomidine were observed while renal function was measured by NGAL concentration. NGAL, as an early biomarker of CSA-AKI, was superior to conventional criteria, and the authors finally concluded that dexmedetomidine could be useful in the prevention of CSA-AKI. Based on the aforementioned, the evidence from cohorts and RCTs supports the renoprotection of dexmedetomidine.

Because meta-analysis is based on collected data, it is impossible to assess the effect of dexmedetomidine on the severity and duration of CSA-AKI beyond included studies. The recent RCT by Zhai et al. [5] was not included because it was published online after our manuscript submission. Additionally, the pooled effect after including this RCT also suggested that dexmedetomidine could decrease the CSA-AKI risk (RR 0.42, 95% CI 0.26–0.69, *P* = 0.0007) without significant heterogeneity (*P*_H_ = 0.75, *I*^2^ = 0%).

In conclusion, we would emphasize that a clue toward but not a definite conclusion of “dexmedetomidine being a promising prevention strategy for CSA-AKI” was drawn, as we summarized in the last sentence of our study.
